# Lipophilic chemical exposure as a cause of cardiovascular disease

**DOI:** 10.2478/intox-2013-0010

**Published:** 2013-06

**Authors:** Harold I. Zeliger

**Affiliations:** Zeliger Chemical, Toxicological and Environmental Research, West Charlton, New York, USA

**Keywords:** cardiovascular disease, environmental disease, heart disease, lipophilic chemicals, toxic chemicals

## Abstract

Environmental chemical exposure has been linked to numerous diseases in humans. These diseases include cancers; neurological and neurodegenerative diseases; metabolic disorders including type 2 diabetes, metabolic syndrome and obesity; reproductive and developmental disorders; and endocrine disorders. Many studies have associated the link between exposures to environmental chemicals and cardiovascular disease (CVD). These chemicals include persistent organic pollutants (POPs); the plastic exudates bisphenol A and phthalates; low molecular weight hydrocarbons (LMWHCs); and poly nuclear aromatic hydrocarbons (PAHs). Here it is reported that though the chemicals reported on differ widely in chemical properties and known points of attack in humans, a common link exists between them. All are lipophilic species that are found in serum. Environmentally induced CVD is related to total lipophilic chemical load in the blood. Lipophiles serve to promote the absorption of otherwise not absorbed toxic hydrophilic species that promote CVD.

## Introduction

Cardiovascular disease (CVD), in the world continues to increase dramatically (Murray *et al.*, 2012; Naghavi *et al.*, 2012; Vos *et al.*, 2012). Worldwide, more than 2.6 million people die from CVD annually (WHO, 2006). In the 20 year period of 1990–2010, deaths from cardiovascular disease across the globe have risen by more than 30 percent (Naghavi *et al.*, 2012). The increases in many epidemic and pandemic diseases, including CVDs, have been attributed to environmental exposures to exogenous toxic chemicals. The World Health Organization estimates that “as much as 24% of environmental disease is caused by environmental exposures that can be averted” and that worldwide, more than 2.6 million people die from CVD annually (WHO, 2006). Recent research has shown that CVD prevalence is increased by exposure to a number of different chemicals. These include persistent organic pollutants (POPs) – polychlorinated bipehenyls (PCBs) (Lind & Lind 2012; Lind *et al.*, 2012a; Ha *et al.*, 2007; Everett *et al.*, 2011; Sjoberg *et al.*, 2013), organochlorine pesticides (OCs) (La Merrill *et al.*, 2013; Lind & Lind, 2012; Lind *et al.*, 2012a; Valera *et al.*, 2012), dioxins and furans (Lind & Lind, 2012; Lind *et al.*, 2012a; Ha *et al.*, 2007; Brown 2008; Everett *et al.*, 2011), polybrominated biphenyl ethers (PBDEs) used as fire retardants (Lind & Lind, 2012; Lind *et al.*, 2012a; Ha *et al.*, 2007) and esters of perfluorooctanoic acid (PFOEs), widely used in cleaning products (Shankar *et al.*, 2012; Min *et al.*, 2012; Holtcamp, 2012); bisphenol A, widely used in the manufacture of plastic food containers and other applications, (Lind & Lind, 2012; Melzer *et al.*, 2010; 2012a, b; Shankar *et al.*, 2012; Bae *et al.*, 2012; Olsen *et al.*, 2012a, b); and phthalates, widely used as plasticizers for polyvinyl chloride, (Singh & Shoei-Lung, 2011; Lind & Lind, 2011; 2012; Olsen *et al.*, 2012a, b), which are exuded from plastics; low molecular weight aliphatic and aromatic hydrocarbons (LMWHCs) and their chlorinated products which evaporate from gasoline, adhesives, paints and household products (ATSDR, 2001; Morvai *et al.*, 1976; Capron & Logan, 2009; Tsao *et al.*, 2011; Xu *et al.*, 2009; Tsai *et al.*, 2010; Kotseva & Popov, 1998; Rosenman, 1979; Rufer *et al.*, 2010); and polynuclear aromatic hydrocarbons (PAHs) which come from primary and secondary tobacco smoke and fuel combustion (Wellenius *et al.*, 2012; Martinelli *et al.*, 2013; Liu *et al.*, 2013). Mechanisms of action have been suggested for some of these chemicals, but to date no one mechanism can account for the cardiovascular toxicity of this diversified group of chemicals which differ in widely in structure, chemical properties and reactivity (Yokota *et al.*, 2008; Toren *et al.*, 2007; Burstyn *et al.*, 2005; Iwano *et al.*, 2005; Mustafic *et al.*, 2012; Wichmann *et al.*, 2013; Brunekreef *et al.*, 2009; Chen *et al.*, 2008).

It is reported here that there is indeed a unifying explanation for the induction of CVD by this diversified group of chemicals. The studies above show that accumulation of all of these chemicals in body serum has been associated with increased incidences of CVD. All these chemicals are lipophilic and all have been shown to accumulate in body serum following exposure to them. It has been previously reported that lipophilic chemicals facilitate the absorption of hydrophilic chemicals across the body's lipophilic membranes (Zeliger, 2003; 2011). It is proposed here that the lipophilicity of these exogenous chemicals induces CVD by permeating lipophilic membranes and thus providing entry for toxic hydrophilic species that would otherwise not be absorbed.

It has been previously shown that mixtures of lipophilic and hydrophilic chemicals are toxic to humans at concentrations that are far below those known to be toxic for the each of the components of such mixtures (Zeliger, 2003; 2011). It has also been previously shown that exposures to the hydrophilic and lipophilic chemicals need not occur simultaneously, but can occur sequentially, with the lipophilic exposure coming first and the hydrophilic exposure occurring some time later, provided that the lipophilic species are still retained in the body (Zeliger *et al.*, 2012). Such a sequential phenomenon has been demonstrated for the induction of type 2 diabetes (Zeliger, 2013) and is believed the case with the induction of CVD.

In the case of CVD, the lipophiles can be long-lived POPs, which once absorbed can remain in the body's adipose tissue for up to 30 years or more and can be transferred to serum (Yu *et al.*, 2011). The lipophiles can also be intermediate lived species, including PAHs, BPA and phthalates, which can remain in the body for days or weeks (Stahlhut *et al.*, 2009; Kessler *et al.*, 2012; Li *et al.*, 2012). Even LMWHCs are retained in body serum for days after absorption (Pan *et al.*, 1987; Zeliger *et al.*, 2012). The serum concentrations of LMWHCs remain more or less in a steady state due to continuous exposure and absorption that replaces quantities lost via metabolism and elimination (Basalt, 2000).

Other lipophilic chemicals that people are constantly exposed to, and that are retained in body serum, include mycotoxins released from mold (Brasel *et al.*, 2004; Bennett & Klich, 2003; Brewer *et al.*, 2013), anti-oxidants and other preservatives added to foods and cosmetics, including triclosan (Queckenberg *et al.*, 2010; Sandborgh-Englund *et al.*, 2006) and, butylated hydroxyanisole (BHA) and butylated hydroxytoluene (BHT) (Surak *et al.*, 1977; Verhagen *et al.*, 1989; Conning & Phillips, 1986), chlorinated derivatives of methane that are the bye products (DBPs) of the disinfection of water by chlorine, including chloroform and the bromo-chloro-methanes (Zeliger, 2011), the chlorinated derivatives of ethane, including 1,1,1-trichloroethane, trichloroethylene, tetrachloroethylene, that arise from cleaning products and contamination of aquifers (Zeliger, 2011), and the presence of pharmaceuticals that are found in drinking water in many cities (Donn, 2008).

It is proposed here that the structure of the lipophile, whether a POP, a plastic exudate, hydrocarbon, mycotoxin, food additive, chlorinated hydrocarbon or pharmaceutical is not the critical point. Rather, it is the lipophilicity and total serum load of lipophilic species that is the determining factor in triggering CVD. Once a steady-state critical dose of lipophile is reached, the body is ripe for sequential attack by hydrophilic species, with the mixture of lipophile and hydrophile able to attack even at low levels of exposure (Zeliger, 2003; 2011, Zeliger *et al.*, 2012).

## Methods

The results presented here is based upon a literature review of numerous published studies, both by this author and others, on the toxic effects of the chemicals involved, including case studies and epidemiologic studies. Health effects noted were, in all instances, diagnosed by appropriate clinical examinations and tests, and chemical analytical data were generated in accordance with accepted protocols.

## Results

### Lipophile – Hydrophile

Most body tissues and cells are coated with lipophilic mucous membranes that serve as a barrier to chemical absorption (Alessio, 1996, Zeliger, 2003). Lipophilic chemicals penetrate mucous membranes much more readily than hydrophilic species (Witte *et al.*, 1995) and mucous membrane barriers serve to protect against absorption of hydrophilic chemicals (Kitagawa *et al.*, 1997). Lipophilic chemicals are routinely used to promote the permeation of hydrophilic species and are used in pharmaceutical delivery systems since most hydrophilic drugs do not penetrate epithelial barriers at rates necessary for clinical usefulness without lipophilic permeability enhancers (Manganaro, 1997; Ghafourian *et al.*, 2010; Pohannish, 2012).

The designation of a chemical as a lipophile, as used here, is based on octanol:water partition coefficients (Kow). Kow is indicative of the relative lipophilic character of a given chemical. It is defined as the logarithm of the ratio of that quantity of chemical dissolved in the n-octanol phase to that dissolved in the water phase of an octanol-water mixture. Species with Kow of 2.00 or higher are considered lipophilic and those with Kow values of less than 2.00 are labeled as hydrophilic (Zeliger, 2003).

As a general rule, hydrophilic chemicals are more acutely toxic than lipophilic chemicals as can be seen from their permissible exposure levels (Pohanish, 2012). The body's lipophilic barriers, however, protect it from penetration by hydrophiles, which are metabolized and eliminated more rapidly than lipophiles. In mixtures of lipophilic and hydrophilic chemicals, the lipophiles facilitate the absorption and retention of hydrophiles as well as the delivery of hydrophiles to organs and systems which they do not reach alone. Mixtures of lipophilic and hydrophilic chemicals produce toxic effects that are not anticipated from the known toxicologies of the individual species (Zeliger, 2003).

### Sequential absorption

As used here, sequential absorption refers to the initial absorption of a lipophilic species onto or into a lipophilic membrane followed by adsorption of a hydrophilic moiety into the lipophilic species to facilitate the absorption of the hydrophile through that membrane. The sequential absorption of the hydrophile can occur at any time from instantaneously to years following the absorption of the lipophile, providing that the lipophile is still present. Persistent organic pollutants such as PCBs, dioxins, furans and organochlorine pesticides are all lipophilic species (Gallo *et al.*, 2011) and are retained in the body for up to 30 years or more (Yu *et al.*, 2011; Gallo *et al.*, 2011; Kouzentsova *et al.*, 2007; Mullerova & Kopecky, 2007; Covaci *et al.*, 2002). Even the PCB congeners that are not long-lived in the body have been found to be present in the body at elevated levels for long periods of time, suggesting continual exposure to these over time (Gallo *et al.*, 2011). More labile exogenous toxic chemicals are metabolized and/or eliminated from the body and require continuous uptake of lipophiles via inhalation of polluted air, dermal contact, or ingestion of tainted food or water to maintain the critical masses necessary to absorb and transport toxic levels of hydorphiles. The concept of sequential chemical absorption has been described for acute attack (Rea, 1992) as well as for chronic attack (Zeliger *et al.*, 2012) and has been shown to account for the induction of type 2 diabetes (Zeliger 2013).

### Low-level effects

Low-level exposures as discussed here are those to concentrations below the published threshold limit values (TLV), permissible exposure levels (PEL), or maximum contamination level (MCL).

It has been previously shown that mixtures of toxic chemicals containing at least one lipophile and one hydrophile produce effects that are not predicted from the known toxicology of the individual species. These effects include attack on organs and systems not known to be impacted by the individual species and low-level toxicity induced by exposures to concentrations far below those known to be toxic by the single chemicals in the mixtures (Zeliger, 2003). The correlation presented here between lipophilic absorption with sequential hydrophilic absorption corroborates well with these findings.

In all the published studies, the levels of lipophiles in the blood are far lower than those known to be acutely toxic for the individual species.

### Total lipophilic load

Total lipophilic load in serum is postulated as responsible for the induction of CVD. As used here, total lipophilic load refers to the total concentration of all exogenous lipophilic chemicals found in serum, without specification of individual chemical species.

### Mechanism of action

Mechanisms by which environmental chemicals trigger cardiovascular diseases have been proposed. These include: include oxidative stress (Babu *et al.*, 2013; Bae *et al.*, 2012; Jaiswal *et al.*, 2012; Hennig *et al.*, 2002) and endocrine distruption (Schug *et al.*, 2011). Until now, however, no single mechanism that accounts for the induction of a broad spectrum of cardiovascular diseases has been proposed. The association with the onset of several different CVDs with exposures to POPs, BPA, phthalates and hydrocarbons, chemicals which differ widely from each other, strongly suggests a lipophile-dependent mechanism for the induction of CVDs

### Lipophilic chemicals associated with CVD

Exposures to POPs, plastic exudates, PAHs and low molecular weight hydrocarbons (LMWHCs) have been found to be associated with CVDs. The POPs include PCBs, OCs, dioxins and furans, PBDEs and PFOEs. Plastic exudates include BPA and phthatlates. LMWHCs include benzene, toluene, ethyl benzene, xylenes, C3–C8 aliphatics, gasoline, chlorinated methanes and ethanes and chlorinated ethylenes. PAHs include the following 17 compounds:

- Acenaphthene
- Acenaphthylene
- Anthracene
- Benz[a]anthracene
- Benzo[a]pyrene
- Benzo[e]pyrene
- Benzo[b]fluoranthene
- Benzo[g,h,i]perylene
- Benzo[j]fluoranthene
- Chrysene
- Dibenzo[a,h]anthracene
- Fluoranthene
- Fluorine
- Indeno[1,2,3-c,d]pyrene
- Phenanthrene
- Pyrene

Though most studies on PAHs have been carried out on benzo[a]pyrene, all 17 of these compounds have been associated with cardiovascular disease and all are environmental pollutant products of the combustion of fuel and tobacco smoke (ATSDR, 1995).

Many different phthalates are used in the manufacturing of phthalates. These include the following 12 compounds (Singh & Shoei-Lung, 2011):

- Diethylhexyl phthalate
- Dibutyl phthalate
- Di-n-pentyl phthalate
- Dicyclohexylphthalate
- Diallyl phthalate
- Diethyl phthalate
- Diisodecyl phthalate
- Di-n-hexyl phthatate
- Diisobutyl phthalate
- Di-n-octyl phthalate
- Diisononyl phthalate
- Diheptyl phthalate

Table 1 lists lipophilic chemicals known to cause CVD and the references for these.

**Table 1:** Lipophilic chemicals known to be associated with cardiovascular disease and the references for these.

CHEMICALS	REFERENCES
**POPs**	
PCBs	Lind & Lind, 2012; Lind *et al.*, 2012; Ha *et al.*, 2007; Ha *et al.*, 2002; Everett *et al.*, 2011; Sjoberg et al., 2013; Lind & Lind 2011
OCs	La Merrill *et al.*, 2013; Lind & Lind, 2012; Lind *et al.*, 2012, Valera *et al.*, 2013
Dioxins/Furans	Lind, 2012; Lind & Lind, 2012a; Brown 2008; Everett *et al.*, 2011; Ha *et al.*, 2007
PBDEs	Lind & Lind, 2012; Lind *et al.*, 2012; Ha *et al.*, 2007
PFOEs	Shankar *et al.*, 2012; Min, 2012; Holtcamp, 2012
**PLASTIC EXUDATES**	
BPA	Lind & Lind, 2011; Melzer *et al.*, 2010; Melzer *et al.*, 2012a; Shankar *et al.*, 2012; Melzer *et al.*, 2012; Bae, et al., 2012; Olsen *et al.*, 2012b; Lind & Lind, 2011
Phthalates	Lind & Lind, 2012; Brown, 2008; Olsen *et al.*, 2012b; Lind & Lind, 2011; Olsen *et al.*, 2012a
**LMWHCs**	
Benzene	Morvai *et al.*, 1976; Kotseva & Popov, 1998
Toluene	ATSDR, 2001; Morvai *et al.*, 1976; Capron & Logan, 2009; Tsao *et al.*, 2011
Xylenes	Morvai *et al.*, 1976; Xu *et al.*, 2009; Tsai *et al.*, 2010; Kotseva & Popov, 1998
Chlorinated solvents	Rosenman, 1979; Rufer *et al.*, 2010
**PAHs**	
	Costello *et al.*, 2013; Yokota *et al.*, 2008; Toren *et al.*, 2007; Burstyn *et al.*, 2005; Iwano *et al.*, 2005; Curfs *et al.*, 2005; Mustafic *et al.*, 2012; Wichmann *et al.*, 2013; Brunkereef *et al.*, 2009; Chen *et al.*, 2008; Wu *et al.*, 2012; Dong *et al.*, 2013; Soghis *et al.*, 2012; Cosselman *et al.*, 2012; Hurt *et al.*, 2012; MMWR, 2009; Bartecchi *et al.*, 2006; Adar *et al.*, 2013; Krishman *et al.*, 2012

### Other lipophiles of exposure

Humans are routinely exposed to many other lipophilic chemicals. Though not reported to cause CVDs, these contribute to the total lipophilic load in body serum and can facilitate the absorption of toxic hydrophiles. These chemicals include: mycotoxins produced by molds and found in wet environments and in contaminated foods (Reddy & Bhoola, 2010; Peraica *et al.*, 1999; Brasel *et al.*, 2004; Brewer *et al.*, 2013); antioxidants put into foods and cosmetics for preservation purposes, including BHA and BHT, (Conning and Phillips, 1986; Verhangen *et al.*, 1989); triclosan, an antibacterial compound widely used in tooth paste, cleaners and other consumer products (Sandborgh-Englund *et al.*, 2006); brominated vegetable oil, used to stabilize citrus-flavored soft drinks (Bernal *et al.*, 1986; Bendig *et al.*, 2013); lipophilic pharmaceuticals, examples of which are statins, taken regularly (Culver *et al.*, 2012; Zeliger, 2012), and pharmaceuticals contained in contaminated drinking water (Donn, 2008).

### Cardiovascular diseases

Exposures to the lipophilic chemicals discussed above have been associated with a broad spectrum of cardiovascular diseases (Humblet *et al.*, 2008). These include: myocardial infarction (Mustafic *et al.*, 2012; Wichmann *et al.*, 2013); atherosclerosis (Whayne, 2011; Lind *et al.*, 2012;); hypertension (La Merrill *et al.*, 2013; Sergeev & Carpenter, 2011; Lind & Lind, 2012; Ha *et al.*, 2009; Valera *et al.*, 2013); coronary heart disease (Shankar *et al.*, 2012; Lind & Lind, 2012); peripheral heart disease (Shankar *et al.*, 2012; Lind & Lind, 2012); ischemic heart disease (Toren *et al.*, 2007; Costello *et al.*, 2013; Burstyn *et al.*, 2005); and cardiac autonomic function (Wu *et al.*, 2012).

## Discussion

The chemicals that are known to cause cardiovascular disease include POPs (PCBs, OCs, PBDEs, dioxins, furans, PFOEs), phthalates, BPA and hydrocarbons. These chemicals come from a variety of chemical classes that include chlorinated and brominated hydrocarbons, esters, ethers, polynuclear aromatic hydrocarbons, mononuclear aromatic hydrocarbons and straight chain aliphatic hydrocarbons. These chemicals differ widely in chemical properties, reactivities and rates of metabolism and elimination from the body.

POPs are long-lived and accumulate in white adipose tissue (WAT) from which they can transfer to the blood and be transported around the body (Yu *et al.*, 2011; Mullerova & Kopecky, 2007; Covaci *et al.*, 2002). Due to the slow rates of metabolism and elimination, POPs can persist in the body for 30 years or longer once absorbed and can build up with time to toxic concentrations (Yu *et al.*, 2011; Gallo *et al.*, 2011). This bioaccumulation of POPs with time over many years accounts for the delayed onset of CVDs following initial exposure.

The lower molecular weight CVD inducing chemicals (phthalates, BPA, PAHs and LMWHCs, can be absorbed at toxic concentrations. Though these are more rapidly metabolized/eliminated, nevertheless, they persist in body serum for days to weeks (Stahlhut *et al.*, 2009; Koch *et al.*, 2004; Li *et al.*, 2012; Pan *et al.*, 1987). Accordingly, short-term toxic concentrations from single exposures to these are fairly rapidly reduced. All of these chemicals, however, are ubiquitous in the environment as air, water or food contaminants, making for fairly continuous absorption and the maintenance of steady-state concentrations in the blood of those who are continually exposed. Such a scenario applies as well to those who take some pharmaceutics on a regular basis and produce fairly constant levels in the blood stream (Zeliger, 2012; Culver *et al.*, 2012).

The chemicals described above, however, have one characteristic in common, they are all lipophiles. Although the exposure levels of these lipophilic species are much lower than their known toxic levels, they are high enough to provide a vehicle for the sequential absorption of toxic hydrophilic species (Zeliger *et al.*, 2012; Zeliger, 2013). It is well known that mixtures of lipophilic and hydrophilic species induce low-level toxic effects and unanticipated points of attack (Zeliger, 2003; 2011), and it is proposed here that combinations of low-level lipophile/hydrophile mixtures act as agents for CVD induction.

Support for this proposal comes from a consideration of other environmental diseases that have been attributed to exposures to these chemicals. POPs exposures have been associated with type 2 diabetes (Zeliger, 2013; Lee *et al.*, 2010; Carpenter 2008); immunological disorders (Hertz-Picciotto *et al.*, 2008; Noakes *et al.*, 2006; Tryphonas, 1998), musculoskeletal disorders (Lee *et al.*, 2007), reproductive interferences (EPA, 2008; Nishijo *et al.*, 2008; Herz-Picciotto *et al.*, 2008), endocrine disruption (Snyder & Mulder, 2001; Colborn *et al.*, 1997), periodontal disease (Lee *et al.*, 2008), neurological disease (Kodavanti, 2005; Patri *et al.*, 2009; Gamble, 2000; White & Proctor, 1997; Burbacher, 1993), neurodevelopmental disorders (Grandjean & Landrigan, 2006; Polanska *et al.*, 2012; Korrich & Sagiv, 2008; Yolton *et al.*, 2011;) and neurodegenerative diseases, including ALS, Alzheimer's and Parkinson's diseases (Parron *et al.*, 2011; Loane *et al.*, 2013; Chen *et al.*, 2013; Steenland *et al.*, 2012; Wang *et al.*, 2011; Dardiotis *et al.*, 2013; Caudle *et al.*, 2012; Weisskopf *et al.*, 2010; Moulton & Yang, 2012; Mayeux & Stern, 2012; Zaganas *et al.*, 2013; Sienko *et al.*, 1990; Vincenti *et al.*, 2012). The onset of many different cancers has been associated with exposures to the chemicals described here. A discussion of environmental causes of cancer, however, is beyond the scope of this presentation. Zeliger 2004 and Zeliger 2011 offer an introduction to this topic. Though not studied as widely as POPs, BPA, phthalates, PAHs and LMWHCs exposures have also been associated with other environmental diseases (Cooper *et al.*, 2009; Lind & Lind, 2012; Liu et al, 2013; Martinelli *et al.*, 2013). The only mechanism that accounts for all these effects is the sequential absorption of lipophiles followed by hydrophiles.

As previously discussed, PAHs, emanating from the combustion of fossil fuels and tobacco, are considered to induce cardiovascular disease. Several of the studies cited have made the association with inhalation of fine particulates rather than with the PAHs (Costello, et. al., 2013; Toren *et al.*, 2007). It has been shown, however, that the toxicity of the particulates is due to the adsorption of the PAHs on the solid particles and the subsequent partitioning from such particles onto and through lipophilic membranes (Yokota *et al.*, 2008). The fine particles serve as vehicles to deliver the PAHs deep into the lungs, where these compounds are absorbed.

It is to be noted that although the literature relating CVD to other exogenous lipophilic chemicals is scanty, both triclosan (Cherednichenko *et al.*, 2012) and mycotoxin exposures (Ngampongsa *et al.*, 2012; Wang *et al.*, 2009) have been associated with CVD. These have been shown to accumulate in serum (Brewer *et al.*, 2013; Queckenberg *et al.*, 2010; Sandborgh-Englund, *et al.*, 2006) and, as such, contribute to total lipophilic load.

## Conclusion

Cardiovascular disease is rising rapidly throughout the world. It is proposed here that this increase is due in large part to increased exposure to exogenous lipophilic chemicals which, though varying widely in structure, toxicology and chemical reactivity, render the body susceptible to attack via subsequent exposure to low levels of hydrophilic toxins that would otherwise not be absorbed. The lipophilic chemicals can be POPs that are metabolized and eliminated slowly, or BPA, phthalates, PAHs, LMWHCs and other lipophilic species that are eliminated from the body more rapidly, but are constantly replenished in the body from polluted air and water and contaminated food. The accumulation of lipophilic chemicals in the body proceeds until a critical level is reached, at which point the body is vulnerable to attack by low levels of toxic hydrophilic chemicals that would otherwise not be toxic. Sequential absorption of lipophiles followed by hydrophiles provides a unified explanation of how low levels of far different environmental pollutants are responsible for the growing pandemic of cardiovascular disease and other environmental diseases. These findings suggest that allowable levels of exposure need to be dramatically lowered and that research be carried out to find ways to help the body eliminate even low levels of serum exogenous lipophilic chemicals.
